# Clay field therapy for complex PTSD: a single-case study with HRV measurement

**DOI:** 10.3389/fpsyt.2026.1815461

**Published:** 2026-08-13

**Authors:** Magali Démurger, Valentino Pomini

**Affiliations:** Institute of Psychology, Université de Lausanne, Lausanne, Switzerland

**Keywords:** autonomic nervous system, clay field therapy, complex post-traumatic stress disorder (CPTSD), exploratory case study, haptic perception, heart rate variability, psychophysiological regulation, SDNN

## Abstract

**Introduction:**

Complex post-traumatic stress disorder (CPTSD) is associated with dysregulation of the autonomic nervous system, measurable through heart rate variability (HRV); however, the impact of sensorimotor approaches on these markers remains poorly documented. This single-case study examines variations in HRV indices across a ten-session weekly protocol of Clay Field Therapy (CFT) in a 58-year-old woman with CPTSD (DSM-5, TEC, TADS-I) and multiple developmental traumas. The CFT process aligns with sensorimotor therapy principles and Herman's three-phase model of complex trauma treatment.

**Methods:**

HRV was recorded continuously using a portable ECG device and analyzed through time-synchronized segmentation across three session phases (trust and emotional stabilization, haptic work, meaning-making), through comparison of initial (3F) and final (3L) three-minute reference windows, and through identification of key autonomic windows. Global functioning was assessed with the WHODAS 2.0 pre- and post-treatment.

**Results:**

Marked between-session HRV heterogeneity emerged alongside structured intra-session and longitudinal organization. Mean SDNN increased 3.7-fold across the protocol (S1: 55.3 ms to S10: 202.2 ms), paralleled by a 3.1-fold increase in mean RMSSD (26.5 ms to 81.1 ms). The three phases showed differentiated autonomic profiles: Phase 1 presented the highest parasympathetic values (RMSSD = 78.2 ms; mean SDNN = 154.4 ms, within normative range); Phase 2 reflected moderate sympathetic activation with preserved vagal modulation (SDNN = 140.9 ms; RMSSD = 60.1 ms); Phase 3 presented the highest mean HR (92.3 bpm) and a corrected mean SDNN of 129.8 ms approaching the lower normative boundary. Session 10 was structurally distinct, exhibiting the highest SDNN and RMSSD of the protocol alongside two temporally organized peaks (SDNN = 460 ms and 647 ms; LF/HF = 18.5 and 12.2) coinciding with HAPTIC-coded centering events. WHODAS 2.0 scores indicated a clinically meaningful improvement in global functional disability (48.1% to 37.1%), particularly in social participation.

**Discussion:**

These findings suggest that CFT induces structured autonomic reorganization in CPTSD, with phase-differentiated profiles and session-level trajectories reflecting progressive regulatory capacity. HRV segmentation across therapeutic phases, combined with behavioral coding of haptic praxes, represents an original methodological framework for examining the psychophysiological mechanisms of clay therapy and provides a foundation for larger-scale controlled studies.

## Introduction

The treatment of complex post-traumatic stress disorder (CPTSD) remains a significant clinical challenge, despite considerable therapeutic advances over recent decades (1, 2). Although trauma-focused psychotherapies represent the current standard of care, their efficacy is limited for a substantial proportion of patients (3, 4). These approaches, often intensive and emotionally demanding, can prove excessively confrontational when emotion regulation capacities are restricted, thereby exposing patients to early trauma reactivation and heightening dropout risk. Shame and guilt affects, prevalent in CPTSD, further compromise the therapeutic alliance and treatment tolerability (5–7). Against this background, premature discontinuation is well-documented: approximately one in five patients drop out of adult psychotherapy overall (19.7%; 8), with rates reaching 20.9% in randomized controlled trials of guideline-recommended PTSD treatments (8) and rising to 50–70% in more complex clinical settings including veterans in residential PTSD programs (9) and populations with developmental trauma histories, where emotion regulation difficulties are prominent predictors of dropout (10, 11). Recent qualitative work has further highlighted that dropout is not solely driven by patient characteristics but also by relational and procedural factors, such as therapist-patient misattunement, inert therapeutic relationships, and rigid protocol adherence (12), factors that are particularly sensitive in CPTSD, given the centrality of relational rupture in its aetiology. These observations have prompted increasing interest in more graduated approaches centered on stabilization and emotional safety (13, 14).

The symptoms of CPTSD reflect alterations in the neurobiological mechanisms governing stress regulation, including dysregulation of the autonomic nervous system (ANS), modifications of the hypothalamic-pituitary-adrenal axis, and disruptions of limbic and prefrontal circuits (15–17). These impairments manifest clinically as persistent hypervigilance, emotional instability, and increased vulnerability to somatic disorders (18). Autonomic regulation can be operationalized through heart rate variability (HRV), an index of ANS flexibility. Reduced HRV, frequently observed in CPTSD, reflects sympathetic predominance and diminished physiological adaptability, and is often correlated with symptom severity (19–21). HRV thus serves as an objective, continuous biomarker particularly well-suited to evaluating the effects of therapeutic interventions on autonomic regulation.

Within this context, mind-body approaches have gained increasing prominence, in that they target bodily regulation mechanisms before trauma memory processing begins (13, 22). By simultaneously engaging the sensory, motor, and symbolic dimensions of lived experience, they contribute to restoring psychophysiological continuity and the sense of self-cohesion (23). Among these approaches, Clay Field Therapy (CFT) represents a concrete clinical expression of these principles.

### Clay field therapy: theoretical foundations and mechanisms of action

**Figure 1:**
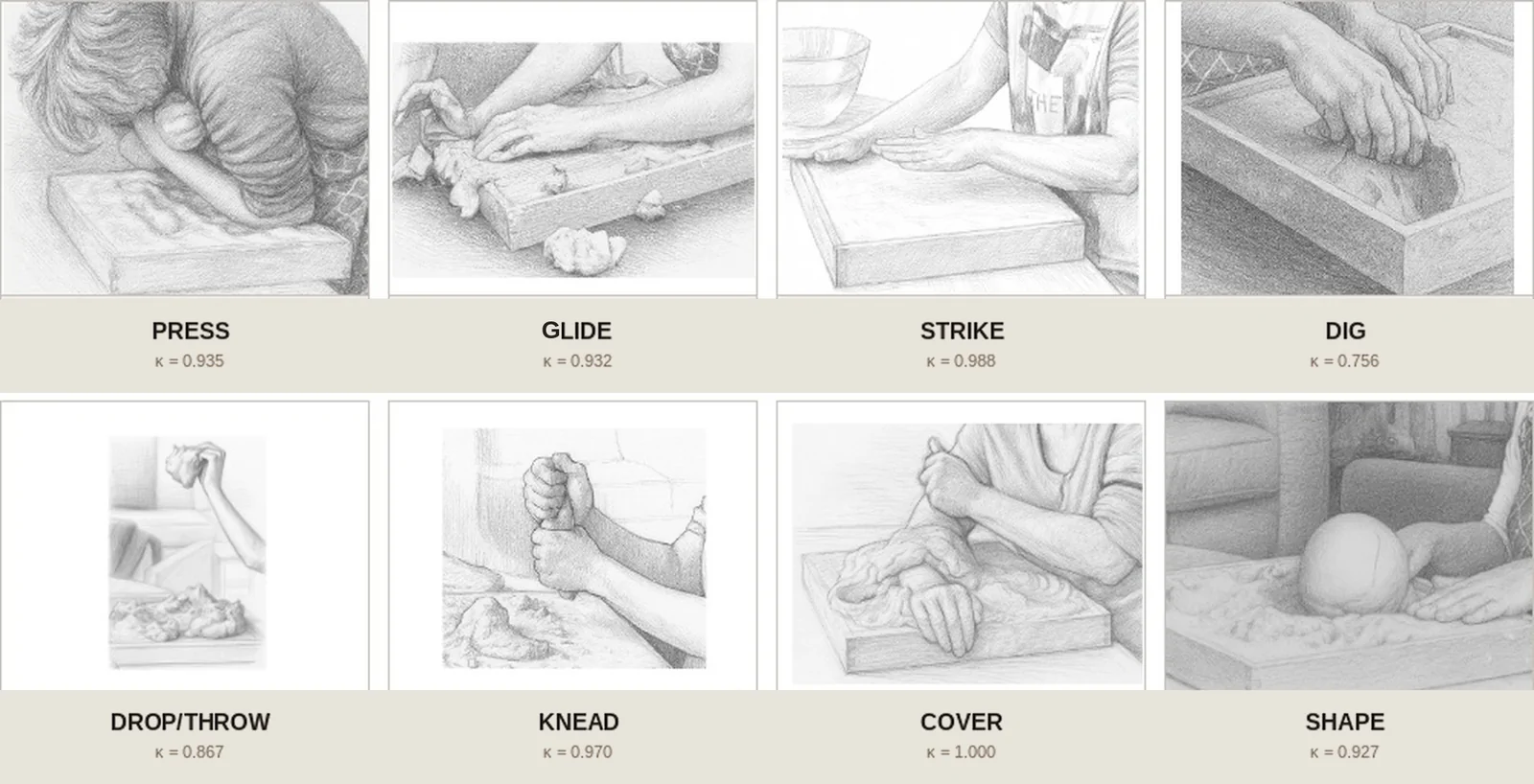
The eight haptic praxes observed during CFT. Schematic representation of the eight generic categories of haptic praxes performed during therapeutic clay work (27): Pressing, Squeezing, Kneading, Gliding, Digging, Covering, Releasing/Throwing, and Shaping. Each praxis is defined by a set of standardized observational descriptors enabling the coding of haptic gestures produced during CFT sessions. HAPTIC, Haptic Analysis and Processing of Therapeutic Interactions with Clay; CFT, Clay Field Therapy (28).

Clay Field Therapy (CFT) is a sensorimotor approach developed by Heinz Deuser in the 1970s, rooted in the existential psychology tradition of Dürckheim (24, 25). Its guiding principle is the free exploration of clay without aesthetic goals or imposed symbolic production: it is the bodily process of contact with the material, not the object produced, that serves as the therapeutic agent (see **Figure 1**). Within the Expressive Therapies Continuum (ETC) framework (26), CFT operates primarily at the kinesthetic/sensory level, which represents the most archaic and somatic stratum of experiential processing, prior to perceptual or cognitive elaboration. .

The central mechanism of action is haptic. Active touch engages sensorimotor loops connecting cutaneous, muscular, and articular receptors to the integrative circuits of the central nervous system (29, 30). This bottom-up regulation unfolds across three progressive levels: sensory regulation is first established through direct contact with the material, activating tactile and proprioceptive afferents; emotional regulation subsequently unfolds through the qualitative modulation of haptic gestures, engaging the limbic circuits implicated in affective processing; and cognitive regulation ultimately emerges from the reflective integration work conducted at the close of each session (31). This process is consistent with the principles of sensorimotor therapy (32) and aligns with Herman’s three-phase model of complex trauma treatment, stabilization, elaboration, and integration (33), as adopted by the ISTSS treatment guidelines (34).

On the empirical level, CFT draws on an established clinical tradition but a still-limited empirical research base. Nan et al. (35) documented the effects of clay manipulation on emotional dysregulation, establishing a neurobiological foundation for the therapeutic properties of this medium. Studies focusing specifically on CFT document effects on affective regulation in ADHD (36) and on adaptive processes in complex grief (37), yet none has measured its effects on autonomic regulation or formalized the observation of gestural behavior through a systematic coding tool. The present study aims to explore precisely this relationship between the organization of haptic praxes and physiological markers of regulation.

This article analyzes the effects of Clay Field Therapy in a patient presenting with CPTSD, based on a ten-session therapeutic protocol conducted in a naturalistic clinical context. To investigate an intervention as nascent and empirically limited as CFT, we opted for a single-case experimental design (SCED). This design, recognized in clinical research on innovative interventions, enables the rigorous study of therapeutic mechanisms in the absence of sufficient preliminary data to justify a randomized controlled trial (38, 39). It also affords the opportunity to examine the intraindividual dynamics of change across treatment, which group designs cannot capture, and represents an increasingly recognized methodological approach in the fields of rehabilitation and clinical neuropsychology (40, 41). The study focuses on the mechanisms of autonomic and emotional regulation engaged during the therapeutic process, exploring the role of haptic praxes in the physiological modulation of stress and in the restoration of stable emotional regulation. The research adopts a mixed-methods approach combining qualitative observation of haptic sequences with quantitative analysis of HRV. This dual level of analysis allows examination of the correspondence between perceptual-motor dynamics and the physiological adjustments observed throughout the therapeutic process. By integrating bodily, perceptual, and physiological dimensions, this study aims to clarify the contribution of haptic praxes to the reorganization of regulatory mechanisms in CPTSD, and to illuminate the psychophysiological foundations of CFT as an integrative therapeutic modality.

## Methods

This study employs a mixed-methods single-case experimental design (SCED). In accordance with established SCED rigor criteria (38, 39), the participant serves as her own control through repeated measurement across time, enabling analysis of the intraindividual dynamics of change across sessions. This design was selected given both the innovative nature of the intervention under investigation and the impossibility of establishing a control group within a naturalistic clinical practice setting, CFT having not yet been the subject of quantitative studies on autonomic regulation. Physiological measures (HRV) serve as the primary dependent variable, assessed continuously and repeatedly throughout the protocol. Qualitative data derived from observation of haptic sequences and functional measures (WHODAS 2.0) serve as secondary variables contributing to the triangulation of findings. A 20-minute structured post-treatment interview was conducted at the end of session 10 to assess the patient’s subjective experience of the therapy.

**Figure 2:**
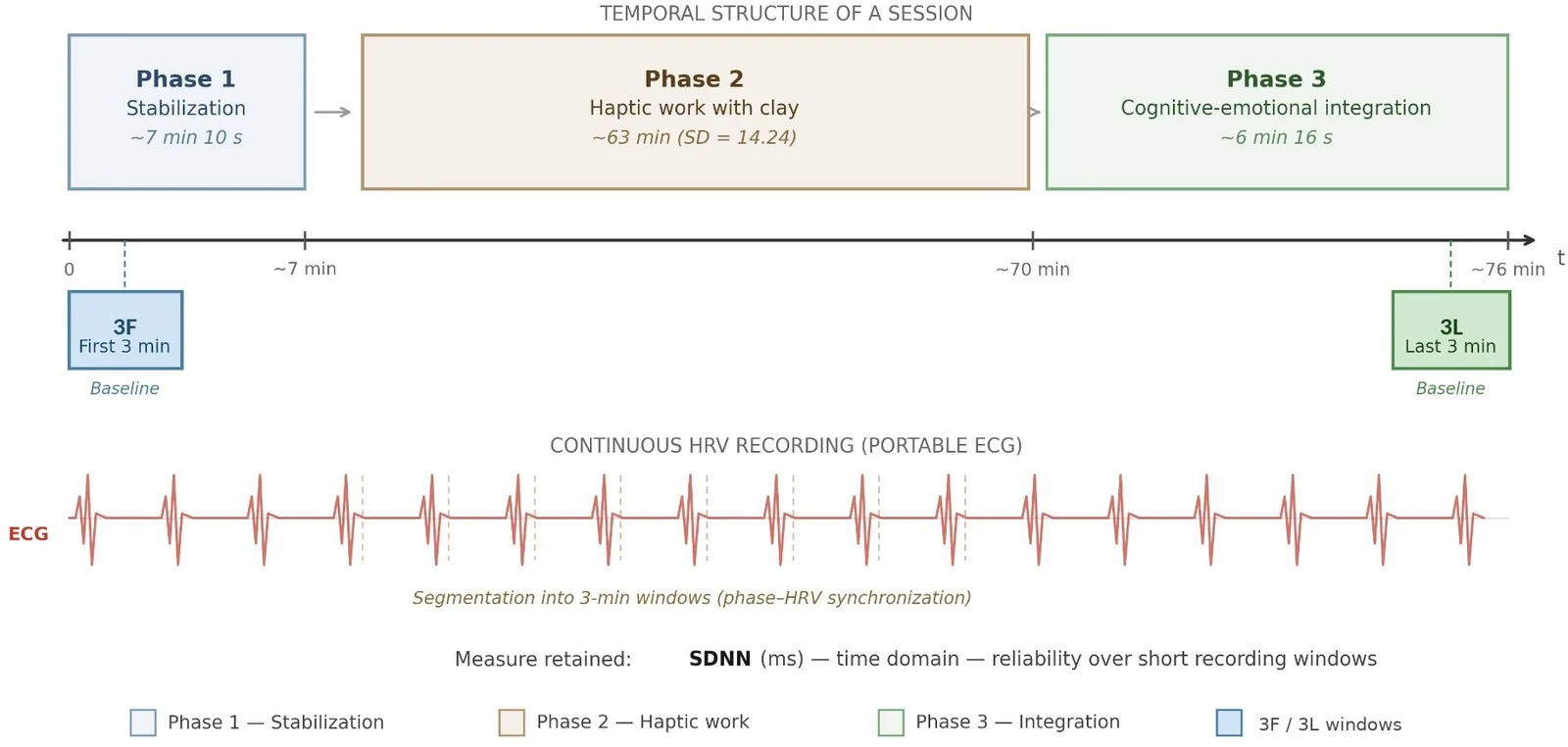
Temporal structure of a CFT session: three phases and reference conditions. Organization of a session into Phase 1 (verbal stabilization; mean duration: 7 min 10 s, SD = 2.9), Phase 2 (haptic work; mean duration: 63 min, SD = 14.24), and Phase 3 (verbal integration; mean duration: 6 min 16 s, SD = 2.7). The 3F (first 3 minutes) and 3L (last 3 minutes) segments used as reference conditions are indicated. CFT, Clay Field Therapy; 3F, first 3 minutes; 3L, last 3 minutes.

Therapeutic protocol. The protocol comprised ten weekly CFT sessions of 60 to 90 minutes each, conducted in a standardized clinical setting by a licensed clinical psychologist trained and certified in CFT practice (see **Figure 2**). Each session began with a preliminary verbal exchange aimed at reviewing the preceding session and the participant’s experiences during the intervening week. The main phase was devoted to haptic work with the clay, followed by a closing period dedicated to verbal elaboration of the experience. During the haptic phase, the therapist remained present in silent attunement, providing minimal verbal prompts only to sustain engagement or support self-regulation when activation emerged. Within the sensorimotor framework underlying CFT, this haptic engagement is understood to support a bottom-up integrative process, in which the processing of bodily sensations arising from contact with the clay precedes verbal articulation (Fisher & Ogden, 2009). All sessions were audio- and video-recorded to enable fine-grained analysis of haptic gestures. Concurrently, a portable device was used to continuously monitor the participant’s HRV.

Variable control. The patient was not receiving any beta-blocker or benzodiazepine treatment, in accordance with the inclusion criteria, thereby preserving the sensitivity of HRV measurements to the most immediate pharmacological influences on autonomic modulation (42). She was, however, receiving a standard-dose SSRI antidepressant, stable for several months prior to the onset of the protocol and maintained without modification throughout the ten sessions. Although SSRIs can theoretically influence autonomic regulation, the meta-analysis by Fiani et al. (43), encompassing 50 studies, concludes that their effects on HRV are non-significant or inconclusive, depending on study design. The stability of the pharmacological regimen across the entire protocol furthermore precludes any differential effect attributable to dosage changes, and the HRV variations observed therefore reflect dynamics occurring within this constant pharmacological context. The patient additionally maintained rigorous consistency in her daily habits, including physical activity, diet, and sleep schedule, and abstained from caffeine for 12 hours prior to each session.

### Case selection

Anne, aged 58, has been married for over 30 years and is the mother of three adult children. Her life trajectory has been marked by a prolonged caregiving role. She had no certified vocational training, having engaged primarily in occasional employment prior to devoting herself to motherhood. She maintains a regular physical activity routine, primarily through walking.

The patient was recruited through a call for participants targeting adults aged 40 to 60 with a CPTSD diagnosis, via a network of partner psychiatrists. She fulfilled all inclusion criteria.

The patient presents a history of complex traumas occurring during childhood, including repeated episodes of psychological maltreatment, physical abuse, neglect, and sexual abuse within the family environment. The diagnosis of complex post-traumatic stress disorder (CPTSD) was established by a psychiatrist according to the criteria of ICD-11 (International Classification of Diseases, 11th Revision; 44). The clinical profile included, beyond standard PTSD criteria, severe disturbances in self-organization and persistent relational difficulties, characteristic of CPTSD as described in the clinical literature (33, 34). This profile was further substantiated by two structured interviews, the Traumatic Experiences Checklist (TEC) (45) and the Trauma and Dissociation Symptoms Interview (TADS-I) (46), the results of which are presented in **Tables 1**, **2**.

**Table 1:** Trauma profile assessed by the traumatic experiences checklist (TEC) (45).

Category	Results
Traumatic exposures	17 events out of 29 items endorsed. Nine rated as having an “extremely significant” impact; five as “significant.”
Perceived social support	Score of zero (0) — complete absence of subjectively perceived support in response to traumatic events.
Identified perpetrators	Immediate family members, neighbors, teachers, healthcare professional.
Types of maltreatment	Emotional neglect (4 perpetrators), emotional abuse (2), physical abuse (1), sexual harassment (4), sexual abuse (2).
Overall clinical profile	Multiplicity, diversity, and chronicity of exposures — profile consistent with CPTSD.

TEC, Traumatic Experiences Checklist; CPTSD, Complex Post-Traumatic Stress Disorder; Self-administered questionnaire (45).

**Table 2:** Traumatic symptoms assessed by the TADS-I (46).

Symptom categories	Childhood	Adolescence	Adulthood
Physical complaints	Headaches; Migraines; Nausea; Enuresis	Persistent	Pain without medical cause; Temporary inability to feel pain
Eating disturbances	—	Eating disorder at age 20 (46 kg/1.61 m)	—
Sleep problems	Difficulty falling asleep; Frequent awakenings; Nightmares; Night terrors; Somniloquy	Difficulty falling asleep; Frequent awakenings; Nightmares	Persistent
Mood changes	Abrupt mood shifts; Intense emotions	Persistent	Persistent; Major depressive episode at age 40; Suicidal ideation without attempt
Anxiety symptoms	Agitation; Repetitive thoughts	Agitation; Repetitive thoughts; Hypervigilance	Persistent
Thought disturbances	Intrusive thoughts; Repetitive frightening images; Flashbacks; Self-destructive behaviors; Psychogenic amnesia	Intrusive thoughts; Repetitive frightening images; Flashbacks; Self-destructive behaviors	Intrusive thoughts
Dissociative symptoms	—	Episodes of depersonalization	Episodes of depersonalization

TADS-I, Trauma and Dissociation Symptoms Interview (46). Semi-structured interview assessing traumatic and dissociative symptoms associated with CPTSD. “—”, symptom absent or not reported for that period. The profile reveals a progressive worsening and complexification from childhood to adulthood.

Prior to initiating the protocol, the patient completed a pre-experimental questionnaire. She rated her general stress level at 3 out of 4, describing herself as frequently tense and anxious. Physically, she reported chronic disabling pain attributed to osteoarticular disorders.

### Measures

#### WHODAS 2.0 — assessment of functional disability

The World Health Organization Disability Assessment Schedule (WHODAS 2.0, full 36-item version) was used to measure the functional impact of health problems on daily life. This instrument assesses six domains: cognition and communication, mobility, self-care, interpersonal relationships, daily life activities, and social participation (47). The patient completed this questionnaire before and after the therapeutic protocol (**Table 3**).

**Table 3:** Functional disability assessment — WHODAS 2.0 (pre- and post-treatment scores).

Domain	Pre-treatment (%)	Post-treatment (%)
Cognition and communication	25.00	18.75
Mobility	31.25	25.00
Self-care	25.00	18.75
Interpersonal relationships	68.75	50.00
Daily life activities	50.00	37.50
Social participation	87.50	62.50
Global functional disability score	48.09	37.11

WHODAS 2.0, World Health Organization Disability Assessment Schedule, full 36-item version. Scores expressed as percentage of functional disability (0% = no limitation; 100% = complete limitation). Social participation was the most severely affected domain at pre-treatment.

#### Post-treatment interview – assesment of subjective experience of the therapy

A 20-minute structured interview was conducted at the end of session 10 to document the patient’s subjective experience of the therapeutic process. Responses were transcribed and analyzed thematically.

#### Portable ECG device and HRV analysis

Autonomic reactivity and motor activity cannot be fully dissociated during haptic engagement, since movement itself modulates autonomic state through respiratory, muscular, and sensorimotor feedback loops. To account for this coupling while preserving temporal comparability, HRV indices were computed over standardized 3-minute windows sampled three times before (3P) and three times after (3D) the haptic phase, when the participant was no longer in contact with the clay. These motor-comparable reference windows allow autonomic variations associated with clay manipulation to be interpreted against a consistent non-haptic baseline.

All sessions were monitored using a portable electrocardiography (ECG) device (eMotion LAB Faros 90, Mega Electronics, Kuopio, Finland) enabling continuous HRV recording. Data were processed using Kubios^®^ HRV software (standard version 3.0.0, 2017). Spectral analysis via fast Fourier transform (FFT) enabled identification of the relative contributions of the sympathetic and parasympathetic branches (48).

Artifact correction. An average correction procedure was applied to all recordings to eliminate abnormal fluctuations in heart rate.

Temporal windows. Each session was divided into three-minute windows to enable precise synchronization between HRV measures and sequences of the therapeutic process.

Establishment of reference values. A reference value was determined from the first three minutes (3F) and the last three minutes (3L) of each session; periods corresponding to verbal exchanges in a seated position. This choice rests on two methodological rationales. First, these segments are structurally homogeneous across the ten sessions, characterized by the absence of hand movement, stable posture, and the presence of verbal exchange, making them controlled conditions comparable to those used in reference studies on HRV in clinical contexts (49). Second, the exclusion of haptic work phases from the reference conditions isolates the influence of manual movements on HRV which would introduce a confounding variable if included in the baseline (50). Longitudinal comparison of the 3F and 3L values thereby enables evaluation of the autonomic state at session entry and exit, independently of the bodily work that separates them.

#### HRV analyses: time-domain and frequency-domain indicators

Heart rate variability (HRV) was analyzed across successive three-minute time windows. In the time domain, SDNN (standard deviation of normal-to-normal intervals) and RMSSD (root mean square of successive differences) were computed as indices of overall autonomic variability and vagal modulation, respectively (51). Although RMSSD is widely used to estimate parasympathetic activity, its reliability decreases in segments shorter than five minutes (20); SDNN therefore provides a more integrative assessment of sympathovagal balance in this context (50). In the frequency domain, the spectral power of the low-frequency (LF: 0.04–0.15 Hz) and high-frequency (HF: 0.15–0.40 Hz) components was estimated using both fast Fourier transform (FFT) and autoregressive (AR) modeling; the LF/HF ratio was retained as an index of sympathovagal balance (51). For the target population (50–59 years), normative SDNN values are approximately 134.9 ± 34.2 ms (49, 52–54). Within-session variability was characterized by the delta (Δ), defined as the range between the minimum and maximum values recorded across each session. Given the sample size (n = 10), all analyses were strictly descriptive; no inferential statistics were performed.

#### AI-assisted HRV data analysis

HRV parameters were extracted from ECG recordings using Kubios^®^ HRV Standard software (version 3.0.0, University of Eastern Finland, Kuopio, Finland) (48). For each session, raw values generated by Kubios^®^ were exported and entered into a Microsoft Excel spreadsheet without prior processing. Only the numerical values of the HRV parameters (Mean RR, Mean HR, SDNN, RMSSD, HRV Triangular Index, TINN), organized by session, phase, and 3F–3L values, were submitted to a large language model (LLM), Claude (Anthropic, claude-3-7-sonnet, 2025). The LLM performed the following operations: (1) tabular organization of raw data by session and phase; (2) positioning of each value relative to age-specific normative reference intervals for the 50–59-year-old population (49, 52–54); (3) identification of outlying values potentially indicative of measurement artifacts; and (4) description of autonomic profiles on a session-by-session basis.

To verify the consistency of the descriptive quantitative outputs (percentages and frequencies), the same anonymized dataset and identical analytical prompts were independently submitted to a second large language model (ChatGPT, OpenAI, GPT-4o, 2024). Outputs from both systems converged, supporting the robustness of the reported descriptive results.

All clinical interpretations, scientific conclusions, and integration of findings within the theoretical framework of the study were conducted exclusively by the principal investigator. The analysis of video data pertaining to gestural praxes involved no AI assistance: HAPTIC coding was performed entirely through direct human observation, in accordance with the procedures defined in the coding manual (27).

### Ethical considerations

This study was conducted in accordance with the ethical principles of the Declaration of Helsinki. The protocol received approval from the Cantonal Research Ethics Committee of Vaud (CER-VD). The participant was provided with detailed information regarding the study’s objectives, procedures, and measures. She gave written, free and informed consent, with the option to withdraw at any time without justification or consequence for her clinical follow-up. Data anonymization was ensured through the use of a fictitious first name and the removal of all identifying information.

## Results

A total of 246 valid 3-minute windows were recorded across the ten Clay Field Therapy sessions (range: 16–31 windows per session). All sessions satisfied the minimum duration criterion for short-term spectral HRV analysis (51). No windows were excluded on technical grounds. Session duration ranged from 48 to 93 minutes (mean = 73.8 min), reflecting the non-directive, client-paced nature of the therapeutic protocol.

Descriptive statistics for the primary HRV indices are reported in **Table 4**. Across the full dataset, the mean RR interval (MRR) ranged from 559.6 ms (S4; HR ≈ 107 bpm) to 863.6 ms (S3; HR ≈ 69 bpm). RMSSD ranged from 26.5 ms (S1) to 81.1 ms (S10). Mean SDNN, an index of total cross-frequency variability, spanned an even wider range, from 41.0 ms (S4) to 202.2 ms (S10), reflecting between-session differences in the magnitude of global autonomic oscillatory activity. The intra-session coefficient of variation of RMSSD (CV RMSSD) ranged from 0.30 (S10) to 1.00 (S6). The RMSSD/SDNN ratio, an index of the vagal contribution to total variability, ranged from 0.515 (S6) to 0.768 (S5); these session-level means nonetheless obscure considerable window-level variability, as detailed in **Table 5**.

**Table 4:** Descriptive HRV statistics by session and autonomic profile.

Session	Duration (min)	Windows (n)	Mean MRR (ms)	Mean RMSSD (ms)	Mean SDNN (ms)	CV RMSSD	RMSSD/SDNN ratio	Mean LF/HF (FFT)	Autonomic profile
S1	48	16	806	26.5	55.3	0.49	0.573	3.73	Paradoxical Opening
S2	72	24	698	29.4	49.7	0.49	0.640	2.73	Low Baseline Tone
S3	87	29	864	74.0	139.1	0.53	0.630	3.94	Unstable Co-activation
S4	93	31	560	30.0	41.0	0.58	0.747	3.05	Sympathetic Defense
S5	93	31	651	54.9	98.8	0.35	0.768	2.95	Moderate Regulation
S6	81	27	737	39.3	114.8	1.00	0.515	4.69	Dissociative Turbulence
S7	72	24	669	32.1	54.6	0.45	0.587	3.82	Low Baseline Tone
S8	78	26	814	55.7	173.0	0.53	0.540	6.89	Emerging Co-activation
S9	66	22	652	27.2	45.7	0.59	0.647	4.78	Late Sympathetic Escalation
S10	48	16	808	81.1	202.2	0.30	0.586	4.43	Integrated Co-activation

MRR, mean RR interval; RMSSD, root mean square of successive differences between adjacent RR intervals (index of vagal tone); SDNN, standard deviation of all RR intervals (total variability); CV, intra-session coefficient of variation (SD/mean); RMSSD/SDNN ratio, proportion of total variability of vagal origin (> 0.75: vagal dominance; 0.50–0.75: sympathovagal balance; < 0.50: predominance of slow non-vagal oscillations); LF/HF = low-frequency to high-frequency ratio (FFT method). Autonomic profiles are descriptive typological labels derived from a four-variable composite pattern analysis (see Section 3.4).

**Table 5:** Key windows (MRR > 900 ms or LF/HF > 10) in sessions 6, 8, and 10.

Session	Window (min)	MRR (ms)	RMSSD (ms)	SDNN (ms)	RMSSD/SDNN Ratio	LF/HF FFT	LF/HF AR	Observed pattern
S6	W1–3 (0–9)	1,087–1,677	84–97	393–624	0.14–0.25	2.7–8.4	2.2–6.5	Extreme bradycardia — slow oscillation dominance (SDNN max = 624 ms)
S6	W4 (11–15)	661	41.8	203	0.21	19.6	38.7	Sympathetic rebound: MRR drop + LF/HF AR dataset maximum
S8	W19–21 (54–63)	1,469–1,489	94–132	623–671	0.15–0.20	12.2–18.2	9.6–17.8	Deep co-activation: SDNN max = 671 ms, RMSSD max = 132 ms
S8	W22 (63–66)	686	15.2	43	0.35	27.6	10.9	Transient reorganization: RMSSD drop despite elevated LF/HF
S10	W4 (11–15)	1,287	128.7	460	0.28	18.5	11.7	Integrated co-activation — sustained RMSSD (★ HAPTIC centering)
S10	W10 (30–33)	1,215	104.3	647	0.16	12.2	10.1	Integrated co-activation — SDNN max S10 (★ HAPTIC centering)

Window duration = 3 minutes. MRR > 900 ms corresponds to HR < 67 bpm. RMSSD/SDNN ratio < 0.25 indicates predominance of slow oscillations relative to vagal tone. ★ HAPTIC, window coinciding with a clinically observed centering event documented in the HAPTIC coding notes (S10 only).

### Intra-session profiles of SDNN and RMSSD

SDNN and RMSSD are presented jointly for each session. SDNN captures total autonomic variability across all frequency bands, whereas RMSSD specifically reflects short-term parasympathetic modulation. Their concurrent examination enables window-by-window identification of whether observed fluctuations represent a global reorganization of autonomic tone or are predominantly driven by a specific vagal component.

**Figure 3:**
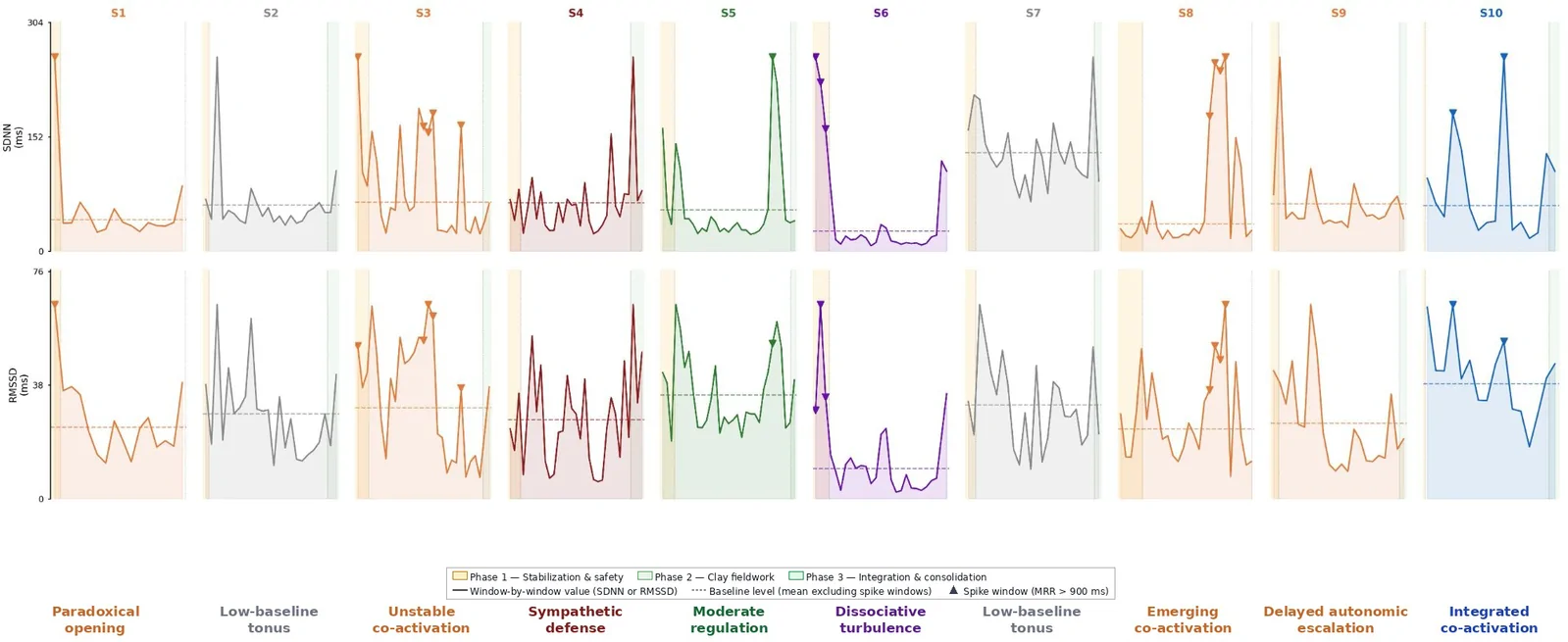
Within-session SDNN and RMSSD profiles across the 10 clay field therapy sessions. CFT, Clay Field therapy; SDNN, Standard Deviation of NN intervals; RMSSD, Root Mean Square of Successive Differences; MRR, Mean RR interval. Each panel displays window-by-window values (3-min epochs) for one session, with SDNN in the upper row and RMSSD in the lower row. Panel color encodes the dominant autonomic profile family named in the session label, not the individual session; sessions sharing a profile share a color. Orange denotes profiles of sympathetic co-activation and instability (S1, S3, S8, S9), gray denotes low-baseline tonus profiles (S2, S7), and the remaining colors denote singular profiles (S4, dark red; S5, green; S6, purple; S10, blue). The dashed line indicates the baseline level, computed as the mean of all windows excluding spike windows. Spike windows (MRR > 900 ms) are marked with a downward triangle (▼) and excluded from baseline computation. Phase backgrounds indicate the three therapeutic phases: Phase 1 — Stabilization & safety (yellow); Phase 2 — Clay fieldwork (session color); Phase 3 — Integration & consolidation (green). Session labels reflect the dominant autonomic profile observed across the session. Two windows were excluded from analysis for technical reasons: Phase 1 of Session 6 and Phase 3 of Session 4. Given the single-case design and exploratory nature of this analysis, no inferential statistics are reported; profiles are presented as descriptive findings.

Across the ten sessions, mean SDNN increased from 55.3 ms (S1) to 202.2 ms (S10) — a 3.7-fold increase — indicating a broadening of total oscillatory variability. Mean RMSSD followed a parallel trajectory, rising from 26.5 ms to 81.1 ms — a 3.1-fold increase. Neither trajectory was monotonic: elevated SDNN and RMSSD values were already observed in S3 (139.1 ms and 74.0 ms, respectively), followed by a return to lower values in S4 (41.0 ms and 30.0 ms) and S7 (54.6 ms and 32.1 ms), before a sustained recovery from S8 onward. The peak SDNN value in S10 (202.2 ms) was accompanied by the highest RMSSD across the entire protocol (81.1 ms), qualitatively distinguishing it from the elevations observed in S3, S5, and S8, in which SDNN markedly exceeded RMSSD (see **Figure 3**).

The RMSSD/SDNN ratio — an index of the vagal contribution to total variability — ranged from 0.515 (S6) to 0.768 (S5). Sessions with higher ratios (S5: 0.768; S4: 0.747) indicated that total variability was predominantly driven by vagal tone. Conversely, sessions with lower ratios (S6: 0.515; S8: 0.540; S10: 0.586) corresponded to configurations in which SDNN markedly exceeded RMSSD, indicating a predominant contribution of slow non-vagal oscillations to total variability. This dissociation between SDNN and RMSSD was particularly pronounced in S6, where a mean SDNN of 114.8 ms coexisted with a mean RMSSD of 39.3 ms; in S8, where SDNN reached 173.0 ms against a RMSSD of 55.7 ms; and in S10, where despite the highest RMSSD in the entire protocol (81.1 ms), the SDNN of 202.2 ms remained markedly elevated, indicating that total variability was driven jointly by vagal tone and high-amplitude slow oscillations.

### Key windows: atypical autonomic configurations

Window-by-window intra-session analysis identified discrete autonomic configurations that deviated markedly from the session background within the temporal dynamics of individual sessions. These key windows were characterized by an atypical concurrent combination of multiple indices (MRR, SDNN, RMSSD, and LF/HF ratio).

Three sessions illustrated configurations of this key windows (**Table 5**). In S6, windows 1–3 (0–9 min) exhibited markedly elevated MRR values (1,087–1,677 ms) alongside SDNN values ranging from 393 to 624 ms and RMSSD/SDNN ratios of 0.14–0.25, indicating total variability dominated by slow oscillations in the context of low vagal tone. Window 4 (9–12 min) marked an abrupt reversal: MRR dropped to 661 ms, and the AR LF/HF ratio reached 38.7 — the highest value recorded across the entire dataset. In S8, windows 19–21 (54–63 min) combined SDNN values of 623–671 ms with RMSSD values of 94–132 ms, both indices reaching their absolute maxima across the entire protocol, before a reorganization at window 22 (RMSSD = 15.2 ms; SDNN = 43 ms). In S10, windows 4 and 10 exhibited MRR values exceeding 1,200 ms with concurrently elevated SDNN (460 and 647 ms) and RMSSD (128.7 and 104.3 ms), and coincided with centering events documented in the HAPTIC coding log (28).

### Analysis of 3P and 3D reference values

The 3P values (session-onset LF/HF ratio) and 3D values (session-end LF/HF ratio) were examined at two levels: inter-session variability, reflecting changes in the LF/HF ratio across the full protocol, and intra-session dynamics, defined as the difference between the initial and final values within each session.

### Distribution of intra-session profiles

**Figure 4:**
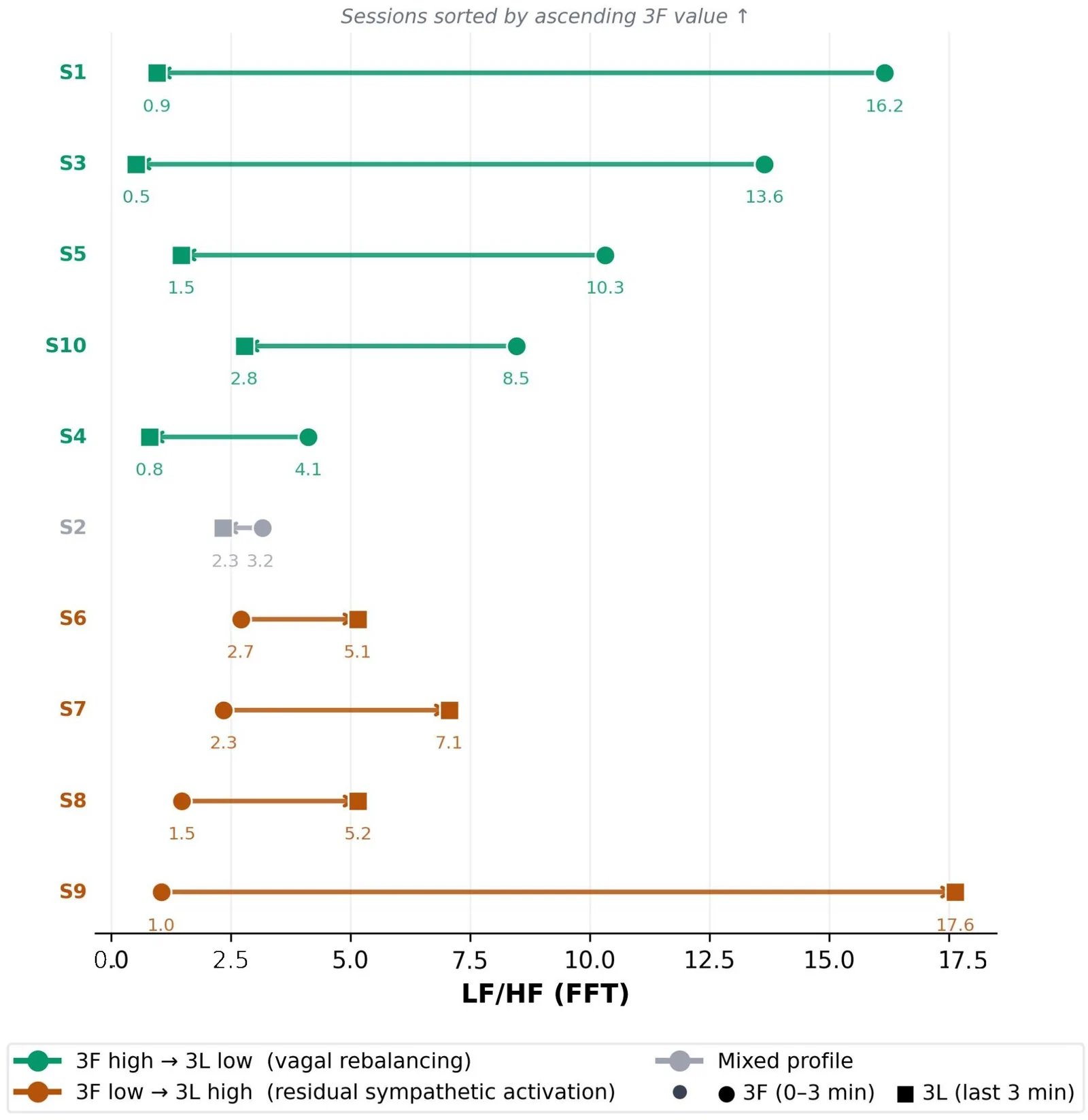
Within-session change in LF/HF ratio (FFT) between the first 3 minutes (3F, circles) and the last 3 minutes (3L, squares) across the 10 CFT sessions, sorted by ascending 3F value. CFT, Clay Field Therapy. Each line represents an individual session (S1–S10), sorted from lowest (top) to highest (bottom) 3F value. Numerical values indicate observed 3F and 3L measurements. Green = sessions with high initial sympathetic dominance (3F) converging toward lower LF/HF at 3L (vagal rebalancing); orange = sessions with low 3F LF/HF diverging toward higher values at 3L (residual sympathetic activation); gray = mixed profile. Given the exploratory nature of this analysis (n = 10 sessions, single-case design), no inferential statistics are reported.

When sessions were ranked by ascending 3P value, two distinct configurations emerged. Five sessions exhibited a vagal rebalancing profile, characterized by a high 3P value and a lower 3D value: S1 (3P = 16.2; 3D = 0.9), S3 (13.6; 0.5), S5 (10.3; 1.5), S10 (8.5; 2.8), and S4 (4.1; 0.8) (see **Figure 4**). The magnitude of intra-session decrease was particularly pronounced in S1 and S3, in which the LF/HF ratio declined by more than tenfold between session onset and offset. Four sessions exhibited a residual sympathetic activation profile, characterized by a low 3P value and a higher 3D value: S6 (3P = 2.7; 3D = 5.1), S7 (2.3; 7.1), S8 (1.5; 5.2), and S9 (1.0; 17.6). Session S9 showed the largest intra-session change in the protocol, with a 16.6-unit increase in the LF/HF ratio. Session S2 exhibited a mixed profile, with minimal variation between the two reference values (3P = 2.3; 3D = 3.2).

### Inter-session trajectory

**Figure 5:**
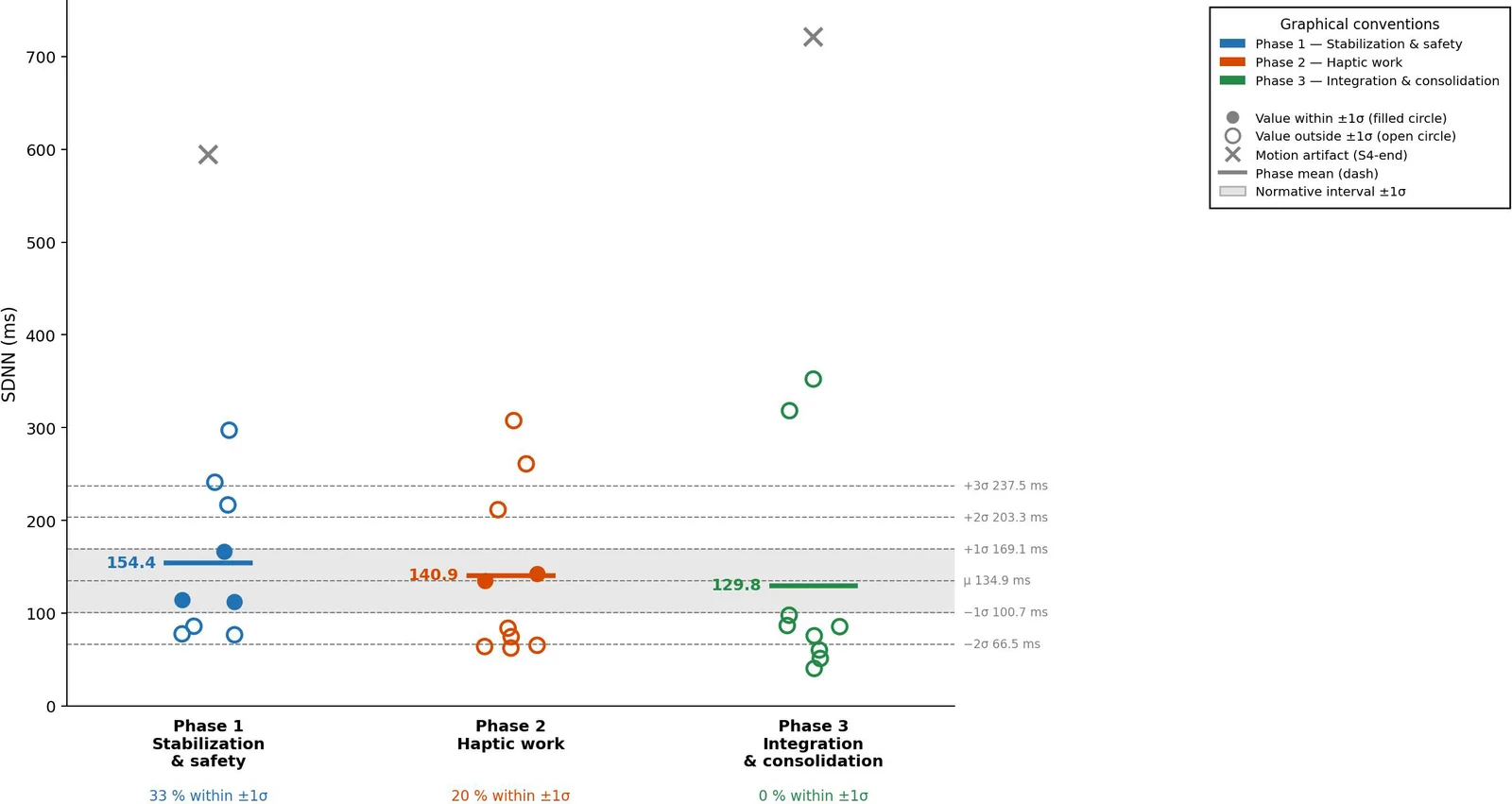
*Distribution of SDNN by therapeutic phase of CFT and relative position with respect to normative reference values (n = 10 sessions).* CFT = Clay Field Therapy. Phase 1: stabilisation and safety (n = 10, mean duration = 7 min 10 s, SD = 2.9); Phase 2: haptic work (n = 10, mean duration = 63 min, SD = 14.24); Phase 3: integration and consolidation (n = 10, mean duration = 6 min 16 s, SD = 2.7). The shaded area represents the normative ±1σ interval for SDNN (134.9 ± 34.2 ms; 44). Horizontal bars indicate the phase mean calculated on valid values only (Phase 1: 154.4 ms, n = 9; Phase 2: 140.9 ms, n = 10; Phase 3: 129.8 ms, n = 9). Open circles indicate values outside the ±1σ interval. Grey crosses indicate values excluded from comparative analyses due to movement artefacts identified during visual inspection of the recordings: S6-start (SDNN = 594.53 ms) and S4-end (SDNN = 720.76 ms).

Examined in chronological order, the distribution of profiles reveals a clear shift between two distinct regimes. Sessions S1 through S5 were predominantly characterized by a vagal rebalancing profile, with high 3P values (range: 4.1–16.2) and low 3D values (range: 0.5–2.3), with the exception of S2, which exhibited a mixed profile (see **Figure 5**). From S6 onward, a complete reversal was observed: sessions S6, S7, S8, and S9 all exhibited a residual sympathetic activation profile, with very low 3P values (range: 1.0–2.7) and progressively increasing 3D values, reaching their peak in S9 (3D = 17.6). Session S10 marked a return to the vagal rebalancing profile (3P = 8.5; 3D = 2.8), representing a terminal inflection point in the protocol trajectory.

### Three therapeutic phases, three autonomic profiles

Analysis of the full set of time-domain HRV parameters reveals a differentiated autonomic profile across three phases of the therapeutic session (trust and emotionnal stabilization, haptic work, meaning-making phase) (**Figure 5**), whose trajectory is not reducible to SDNN alone. The phases are distinguished by their respective mean durations: Phase 1 = 7 min 10 s (SD = 2.9), Phase 2 = 63 min (SD = 14.24), Phase 3 = 6 min 16 s (SD = 2.7).

### Phase 1: stabilization and safety

Phase 1 presents the highest RR intervals of the protocol (MRR = 856.6 ms; HR = 74.1 bpm) and the highest parasympathetic modulation values (RMSSD = 78.2 ms). The mean SDNN, calculated on the nine valid values after exclusion of S6-onset (SDNN = 594.5 ms, a constellation incompatible with a recording of sufficient quality), is 154.4 ms, within the normative interval ±1σ (134.9 ± 34.2 ms; 44). Inter-session variability remains nonetheless considerable: 33% of sessions fall within the ±1σ interval (S2: 114.6 ms; S5: 166.6 ms; S9: 112.7 ms), 33% below −1σ (S4: 77.8 ms; S7: 86.0 ms; S8: 76.8 ms), and 33% above +2σ (S1: 241.1 ms; S3: 297.2 ms; S10: 216.7 ms).

### Phase 2: haptic trauma work

Phase 2 presents a mean HR of 86.8 bpm and a mean MRR of 701.4 ms. The mean SDNN, calculated on the ten valid values, is 140.9 ms; 20% of sessions fall within the ±1σ interval (S4: 142.4 ms; S5: 135.3 ms), 50% below −1σ (S1, S2, S6, S7, S9), and 20% above +3σ (S8: 307.6 ms; S10: 261.0 ms). The mean RMSSD is 60.1 ms, with a median of 45.9 ms, and individual values ranging from 25.1 ms (S1) to 170.8 ms (S4).

### Phase 3: meaning-making phase

Phase 3 records the highest mean HR of the protocol (92.3 bpm) and the shortest RR intervals (MRR = 657.2 ms). On the nine valid values retained after exclusion of S4-end (SDNN = 720.8 ms; RMSSD = 869.2 ms, values inconsistent with a clean recording), the mean SDNN is 129.8 ms, a value that remains within the normative ±1σ interval while approaching its lower boundary (100.7 ms). The mean RMSSD (152.1 ms) remains influenced by the extreme value of S2-end (150.0 ms); the corresponding median is 43.1 ms.

This phase is characterized by the highest inter-session dispersion of the protocol: no individual value falls within the ±1σ reference interval, and three sessions exceed the +3σ threshold (> 237.5 ms), namely S2, S4, and S10. These extreme values are not interpretable in a uniform manner. Session 4 was excluded for the reasons detailed above. Session 2 presents a SDNN of 318.3 ms with RMSSD of 150.0 ms; in the absence of formal artifact markers, these values are retained but interpreted with caution.

Session 10 is structurally distinct. Its Phase 3 SDNN of 352.3 ms represents a continuation of a coherent session-level profile rather than an isolated outlier, as S10 presents the highest mean SDNN (202.2 ms) and mean RMSSD (81.1 ms) of the entire protocol. In contrast to the eight remaining sessions, whose Phase 3 mean HR ranges from 81.1 to 102.4 bpm (M = 92.4 bpm) and mean MRR from 586 to 740 ms, S10 is the only session whose Phase 3 presents an HR below 80 bpm (73.7 bpm), an MRR above 800 ms (814.5 ms), and an RMSSD exceeding the short-duration reference value (92.9 ms vs. 42 ± 15 ms; 47). This profile constitutes the only Phase 3 profile oriented toward parasympathetic dominance in the entire protocol, contrasting with the systematic sympathetic predominance observed at session closure across all preceding sessions.

Taken together, these parameters describe a three-stage trajectory: globally elevated entry values in Phase 1, convergence toward sustained sympathetic activation in Phase 2, and a Phase 3 whose mean SDNN of 129.8 ms indicates a return toward the lower boundary of the normative interval, while nonetheless concealing a persistent inter-session polarization.

### Session 10: autonomic co-activation and haptic continuity

Session 10 exhibited a profile of integrated autonomic co-activation, characterized by SDNN values exceeding +3σ at windows 9–12 min (460 ms) and 27–30 min (647 ms), an FFT LF/HF ratio reaching 18.5 and 12.2, respectively, and an RMSSD/SDNN ratio of 0.31 across the clay work phase. This indicates that extreme total variability was accompanied by a sustained vagal contribution, precluding sympathetic activation as the sole determinant.

**Figure 6:**
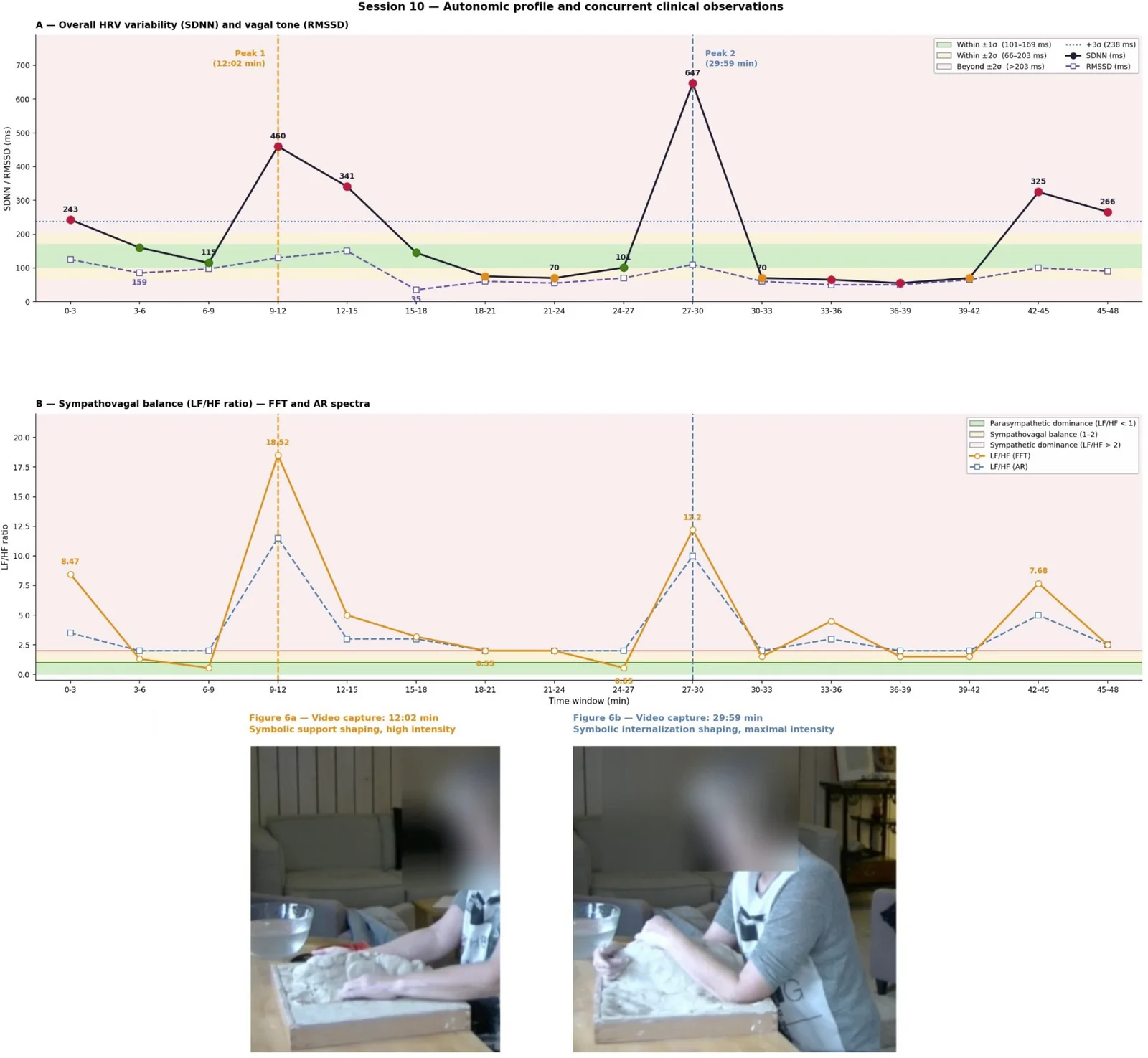
Autonomic profiles and haptic engagements in session 10: window-by-window analysis. Panel **(a)** Window-by-window evolution of global cardiac variability (SDNN, ms, solid line) and vagal tone (RMSSD, ms, dashed line) during session 10. Colored bands indicate normative reference intervals: green (± 1σ, 101–169 ms), orange (± 2σ, 66–203 ms), and red (beyond ±2σ, > 203 ms); the horizontal dotted line demarcates the +3σ threshold (238 ms). Vertical dashed lines mark the two major autonomic peaks: Peak 1 (12:02 min, orange line) and Peak 2 (29:59 min, blue line). Panel **(b)** Evolution of the LF/HF ratio computed by FFT spectral analysis (orange line, circles) and AR analysis (blue dashed line, squares) for the same time windows. Colored bands delimit zones of parasympathetic predominance (LF/HF < 1, green), sympathovagal balance (LF/HF 1–2, orange), and sympathetic predominance (LF/HF > 2, red). Panels **(a, b)** — Video frames synchronized to the two autonomic peaks (28). Panel **(a)** (12:02 min): high-intensity symbolic support shaping, coinciding with Peak 1 (SDNN = 460 ms, LF/HF FFT = 18.52). Panel **(b)** (29:59 min): maximum-intensity symbolic internalization shaping, coinciding with Peak 2 (SDNN = 647 ms, LF/HF FFT = 12.20). Faces have been anonymized in accordance with ethical requirements.

At the haptic level, the session proceeded without gestural discontinuity, the successive praxes forming a coherent sequence throughout the interaction with the clay (coded with the HAPTIC tool; 58). The patient began with low-intensity pressing (approximately 1 minute), followed by progressively increasing sliding movements (approximately 4 minutes), and then alternating striking and sliding gestures of medium-to-high intensity (approximately 3 minutes). From minute 7:49, she engaged in shaping a symbolic form, described by the patient as a mountain and experienced as a source of peace and comfort. This symbolic form was shaped continuously through the end of the clay work phase at minute 44 (see **Figures 6a, b**). This haptic progression is what structured the intra-session autonomic profile: the transition into shaping coincided with the first SDNN peak (window 9–12 min), and the intensification of shaping with the second (window 27–30 min).

Epoch-by-window analysis revealed a temporally organized pattern structured around two symmetric sequences, in which a window of reduced variability with parasympathetic predominance immediately preceded each peak. Windows 6–9 min and 24–27 min exhibited low SDNN values (100–114 ms, < −1σ) associated with an FFT LF/HF ratio below 1 (0.35–0.55), followed respectively by the peak at 9–12 min (SDNN = 460 ms; FFT LF/HF = 18.5; high-intensity HAPTIC coding) and the peak at 27–30 min (SDNN = 647 ms; FFT LF/HF = 12.2; maximum-intensity HAPTIC coding, both hands in contact with the clay mass). These two peaks encompassed the highest FFT LF/HF values recorded across the entire protocol. Windows 0–3 min and 42–45 min exhibited an intermediate profile: SDNN exceeding +2σ with FFT LF/HF above 2, but AR LF/HF below 5, indicating sympathetic predominance of lower intensity (see **Figures 6a, b**).

This temporally organized pattern stands in contrast to the configurations observed in S6 and S8. In S6, the elevated SDNN peak (W4, SDNN = 203 ms; FFT LF/HF = 19.6) occurred as an immediate response to the initial dissociative bradycardia, with no discernible temporal organization thereafter and no identifiable haptic correlate. In S8, the elevated SDNN peaks (W19–21, SDNN = 623–671 ms) appeared abruptly in mid-session, without a preparatory sequence or documented haptic transition. The RMSSD/SDNN ratio computed over the clay work phase further substantiates this distinction: 0.19 in S8, 0.31 in S10, and 0.38 in S6. The value obtained in S8 indicates that elevated total variability was predominantly carried by slow non-vagal components, whereas the value in S10 reflects a sustained vagal contribution within the context of globally elevated variability.

Given the complexity and volume of the HRV dataset, a descriptive label was associated with each session to support the interpretation of the results. These labels emerged inductively during the descriptive analysis and are intended as reading aids highlighting the specific autonomic features of each session. They do not constitute theoretically grounded categories and carry no taxonomic or clinical status.

### Functional improvements

Pre-treatment assessment with the WHODAS 2.0 revealed a global functional disability level of 48.09%, reflecting substantial impairment in daily functioning prior to the intervention. Domain-specific analysis indicated predominant impairment in the relational and social spheres, a profile consistent with observations by Pearson et al. (55) in patients with PTSD.

At treatment completion, reassessment revealed a reduction in total functional disability to 37.11%, representing an improvement of more than 11 percentage points. Although the minimal clinically important difference (MCID) for the WHODAS 2.0 remains context-dependent and has not been universally validated by the WHO (56), this magnitude of change exceeds values reported in PTSD populations studied to date, suggesting a clinically meaningful functional improvement. This improvement was particularly pronounced in the social participation domain, which had been the most severely affected at baseline.

On a qualitative level, data gathered during the post-treatment interview indicated a reduction in social avoidance behaviors, an enhanced capacity to decline requests, and an increase in self-initiated external activities. The patient also reported greater ease in social interactions, without feeling obligated to extend conversations.

## Discussion

The results of this exploratory case study indicate that Clay Field Therapy (CFT) produces measurable and structured changes at two complementary levels: daily functioning, assessed by the WHODAS 2.0, and autonomic regulation, documented through continuous HRV measurement. These two levels do not constitute independent parallel effects; rather, they are interpretable as expressions, at different scales of observation, of a single process of reorganization in the coupling between the organism and its environment.

The reduction in overall disability rate from 48.09% to 37.11%, combined with a 3.7-fold increase in mean SDNN and a 3.1-fold increase in mean RMSSD across the full protocol, indicates that CFT produces a therapeutic effect operating simultaneously on autonomic regulation and daily functioning. SDNN reflects total autonomic variability; RMSSD is specifically sensitive to efferent vagal tone. Their coupled increase signifies that the broadening of total variability is driven by a restoration of vagal tone rather than by an amplification of slow sympathetically driven oscillations, a configuration that would instead characterize progressive dysregulation. This constitutes the signature of restored autonomic flexibility: the capacity of the nervous system to modulate its activation in response to contextual demands rather than to sustain a tonic defensive posture. The longitudinal trajectory of the 3P/3D dynamic further supports this interpretation at a higher level of organization: the progression from a profile of intra-session vagal rebalancing in the early sessions, to a phase of increasing mobilization culminating in S9, followed by a return to a regulated profile in S10, describes a progressive and directional autonomic reorganization across the full protocol, not a random fluctuation. It is the convergence of these indicators that makes sense of what the WHODAS documents: the participant does not report an attenuation of distress, but a transformation of her relationship to the social environment, specifically the capacity to initiate, decline, and engage without the constraint of reciprocity. These results constitute the first continuous psychophysiological corroboration of CFT’s effects, complementing the data reported by Hölz-Lindau (36) on affective regulation in ADHD and the phenomenological transformations documented by Basoglu (37) in complicated grief, thereby establishing a bridge between the subjective experience of therapeutic change and its measurable autonomic correlates.

The three-phase structure of individual sessions provides a complementary level of analysis in relation to the phased model of trauma processing, the international clinical consensus for the treatment of complex PTSD operationalized by Ogden et al. (57) and transposed to CFT by Elbrecht (58, 59) on the basis of her clinical observations. The HRV data allow examination of whether this organization yields a measurable autonomic correlate, and the findings resist a straightforward confirmatory reading.

Phase 1 presents a parasympathetic-dominant profile, with the highest protocol-level values for MRR (856.6 ms), RMSSD (78.2 ms), and the lowest mean HR (74.1 bpm), consistent with a state of somatic availability. The considerable inter-session variability, of which the necessary exclusion of S6-onset (SDNN = 594.5 ms) is symptomatic, nonetheless indicates that initial contact with the therapeutic setting does not produce a uniformly regulated state.

Phase 2 reflects moderate sympathetic activation with preserved vagal modulation (HR = 86.8 bpm; MRR = 701.4 ms; RMSSD = 60.1 ms; SDNN = 140.9 ms). The direction of this autonomic mobilization is consistent with the processing of traumatic material, yet its amplitude, maintained within the normative range, distinguishes this activation from a disorganizing state.

Phase 3 presents the most striking profile. Its pattern of sympathetic predominance, characterized by the highest mean HR of the protocol (92.3 bpm), the shortest MRR (657.2 ms), a mean RMSSD of 43.1 ms following exclusion of S4, and a corrected SDNN of 129.8 ms approaching the lower normative boundary (100.7 ms; 44), corresponds to no common representation of closure or decompression. The marked inter-session polarization of RMSSD in Phase 3 further reveals a degree of heterogeneity obscured by central tendency measures, reflecting deeply individualized integration trajectories across sessions. Of note, S10 presents the only Phase 3 profile oriented toward parasympathetic dominance in the entire protocol (HR = 73.7 bpm; MRR = 814.5 ms; RMSSD = 92.9 ms). In the sense intended by Ogden et al. (57), integration does not designate the resolution of a state but its active incorporation into a new regulatory configuration, a process that these data place as still ongoing at session closure and whose horizon extends beyond the temporal boundary of the session itself.

The concept of the window of tolerance, introduced by Siegel (60) and operationalized clinically by Ogden et al. (57) within the framework of sensorimotor psychotherapy, designates the zone of autonomic activation within which the processing of emotional and somatic information remains possible without tipping into hyperactivation or hypoactivation. Levine (61, 62) extended this framework by emphasizing that the capacity to traverse states of intense activation without disorganization is itself an indicator of acquired regulation, distinct from the mere absence of activation. In complex PTSD, this window is structurally narrowed: low-intensity stimuli suffice to trigger defensive responses that short-circuit integrative processing (63). Analysis of key windows provides here direct psychophysiological evidence of this dynamic, at a temporal resolution unattainable by conventional assessment instruments.

The atypical configurations of S6 illustrate in a particularly striking manner the lower boundary of this window. Windows 1 to 3 (0–9 min) exhibit MRR values reaching 1,677 ms with RMSSD/SDNN ratios of 0.14 to 0.25, a profile characteristic of vagal hypoactivation in a context of extreme total variability, consistent with a dissociative response upon engagement with the therapeutic setting. The abrupt reversal in window 4 (MRR = 661 ms; AR LF/HF = 38.7, the maximum value of the entire protocol) captures a precipitous shift toward sympathetic hyperactivation: the nervous system transitions abruptly between two opposing defensive states, with no access to the zone of integrative processing. This sequence is the psychophysiological transcription of what Siegel (60) describes as oscillation between hypo- and hyperarousal without access to the optimal window, the hallmark of complex trauma.

S8 reveals a different configuration, occurring mid-session: windows 19 to 21 (54–63 min) combine SDNN values of 623 to 671 ms with RMSSD values of 94 to 132 ms, the absolute maximum values of the entire protocol, before an abrupt collapse in window 22 (RMSSD = 15.2 ms; SDNN = 43 ms). The magnitude of this transition within a single three-minute window, absent any preparatory sequence or identifiable haptic correlate, contrasts sharply with the organized logic of S10 and illustrates what Siegel (60) describes as an unstructured excursion beyond the window: the nervous system mobilizes extreme activation resources without a sensorimotor anchor to structure its navigation. This observation clarifies a key interpretive point: the regulatory effects associated with CFT do not arise from the mere physical contact of the hands with the clay, but from the active coupling between manual engagement and the patient’s felt experience. Haptic work is not a passive resting on the material but an intentional engagement in which sensorimotor activity and subjective resonance sustain each other.

It is precisely this feature that distinguishes S10. The two symmetric sequences documented, each comprising a preparatory window with predominant parasympathetic tone (SDNN < 100 ms; FFT LF/HF < 1) immediately followed by a peak of extreme variability (SDNN = 460 and 647 ms) coinciding with major haptic transitions, describe a nervous system that organizes its excursions beyond the optimal zone rather than undergoing them passively. The maintenance of an RMSSD/SDNN ratio of 0.31 throughout these peaks indicates that vagal modulation remains operative even at levels of total variability that, in S6 and S8, were accompanied by a collapse of this contribution. In the sense articulated by Siegel (60), Ogden et al. (57), and Levine (62), this constitutes the signature of an expanded window of tolerance: not the absence of intense excursions, but the capacity to traverse them with regulation sustained. The inter-session progression from the disorganized defensive oscillation of S6 to the structured, vagally supported dynamics of S10 constitutes a trajectory of window-of-tolerance expansion documented in real time, a phenomenon that clinical models postulate but that few studies have been able to measure with this degree of resolution. The interpretation of the results at session 10 must be tempered by an important methodological consideration: this session marked the end of the research protocol, not the end of the participant’s therapeutic trajectory. The observed autonomic and clinical patterns should therefore be understood as reflecting a specific moment within an ongoing process, rather than as evidence of therapeutic resolution.

Several strengths deserve mention. First, the study addresses a gap in the literature on bottom-up therapeutic processes in trauma treatment, a dimension that has received limited empirical attention. Second, the single-case experimental design integrates autonomic, clinical, and functional measures, supporting the internal validity of the findings. Third, the analysis operates at a dual temporal resolution, articulating within-session regulatory moments with the broader therapeutic trajectory, and thereby captures dynamics typically separated in the literature. Finally, the study operationalizes a conceptual articulation between haptic engagement and felt experience that has received limited attention in the trauma literature, opening a line of inquiry at the intersection of sensorimotor psychotherapy and haptic perception research.

This study carries the limitations inherent to its design. A single case in a naturalistic context is appropriate to its exploratory objectives and to the investigation of poorly documented therapeutic processes (38), but precludes any causal inference. The postural and motor confounding factors inherent in haptic work, together with the absence of a control group and of standardized PTSD symptom measures alongside the WHODAS functional assessment, call for replication on larger samples combining HRV with repeated symptom-based evaluation throughout treatment, as recently implemented in trauma-focused paediatric protocols (64). The coupling between haptic praxis organization and autonomic dynamics demonstrated here through the window-by-window analysis of S10 opens new research directions regarding the relationship between embodied symbolization and autonomic nervous system regulation, including the question of whether autonomic reorganization translates into corresponding symptomatic change.

Although the exploratory single-case design of this study precludes firm clinical recommendations, the specific patterns documented here carry implications for how CFT might be positioned in the treatment of CPTSD. The distinction between the disorganized defensive oscillation observed in S6 and the structured, vagally sustained excursions of S10 suggests that the therapeutic value of CFT lies not in the suppression of intense autonomic activation, but in the progressive emergence of a capacity to traverse such activation with regulation preserved. This mechanism is precisely the one that trauma-focused psychotherapies assume to be available when they initiate exposure-based work, yet which is structurally compromised in CPTSD patients with a narrowed window of tolerance. In this sense, CFT may be particularly relevant for patient profiles in whom trauma-focused psychotherapies have been judged premature, such as those presenting with severe emotion dysregulation, high dissociative load, or elevated suicidality. The documentation of a measurable trajectory of window-of-tolerance expansion, independent of verbal processing of traumatic material, further suggests that CFT could function as a preparatory modality restoring the regulatory substrate on which subsequent trauma-focused work depends. Whether CFT should be conceived as an autonomous therapeutic modality, as a preparatory intervention, or as a concurrent regulatory support during more confrontational phases of trauma-focused psychotherapy remains an open empirical question that future controlled studies should address. A related implication concerns therapeutic fidelity: the finding that regulatory effects arise from the active coupling between manual engagement and felt experience, rather than from mere physical contact with the clay, indicates that any adaptation of the protocol which treats haptic work as a passive sensory activity would compromise its active principle.

## Conclusion

This exploratory study demonstrates that continuous HRV measurement, coupled with session-by-session haptic coding, reveals an organization of the therapeutic process that remains invisible to aggregated pre-post assessment protocols. The autonomic heterogeneity characteristic of complex PTSD does not emerge here as methodological noise but as an organized signal, detectable with sufficient temporal resolution. This observation raises a question that extends beyond the present case: instruments centered on symptom reduction likely measure a downstream effect of a transformation whose mechanisms they do not capture. The coupling between haptic praxis and autonomic dynamics demonstrated in S10 further opens a hypothesis specific to clay-based therapeutic work that this material may function as an organizer of autonomic regulation, a function that existing models of body-oriented psychotherapy have yet to formalize. These results support a process-oriented, multi-level approach to the evaluation of trauma psychotherapies, in which continuous measurement of autonomic dynamics occupies a central role that remains to be consolidated through studies on larger samples.

## Data Availability

The raw data supporting the conclusions of this article will be made available by the authors, without undue reservation.
